# A new indicator for the Kunming–Montreal Global Biodiversity Framework: Capturing non-monetary benefit data from access and benefit-sharing agreements

**DOI:** 10.1093/biosci/biae132

**Published:** 2025-02-19

**Authors:** Genuar Nunez-Vega, Lorenz Christian Reimer, Jörg Overmann, Amber Hartman Scholz

**Affiliations:** Science Policy and Internationalization Department, Leibniz Institute DSMZ, Technical University of Braunschweig, Braunschweig, Germany; Department of Bioinformatics, Leibniz Institute DSMZ, Braunschweig, Germany; Leibniz Institute DSMZ, Technical University of Braunschweig, Braunschweig, Germany; Department of Science Policy and Internationalisation, Leibniz Institute DSMZ, Braunschweig, Germany

**Keywords:** biodiversity, indicator, access and benefit-sharing, Nagoya Protocol, Convention on Biological Diversity

## Abstract

The 2022 Kunming–Montreal Global Biodiversity Framework (KMGBF) moves international efforts to conserve biodiversity into a quantitative era. Fair and equitable benefit-sharing is one of the three objectives of the Convention on Biological Diversity, which means that to achieve the KMGBF, its parties will need to begin quantifying the benefits received from access and benefit-sharing (ABS). This mandate represents a big challenge as countries will need to begin to measure both monetary and non-monetary benefits from ABS agreements. Non-monetary benefits, in particular, can be more difficult to measure than monetary benefits, resulting in lower scientific understanding and integration of scientific results into national policy choices. In the present article, we propose a new methodology to deliver data to the KMGBF on non-monetary benefit-sharing indicators using scientific publications that cite ABS permits and put forth recommendations for improving the visibility of non-monetary benefits.

The Kunming–Montreal Global Biodiversity Framework (KMGBF) was adopted at the fifteenth meeting of the Conference of the Parties to the Convention on Biological Diversity (CBD) in December 2022. It sets out four goals for 2050 and 23 targets for 2030 with the purpose of fully implementing the three objectives of the CBD in a balanced manner (COP CBD 2022a). Taken together, the goals and targets of the KMGBF will contribute to the full and balanced implementation of the three objectives of the CBD: the conservation of biological diversity, the sustainable use of its components, and the fair and equitable sharing of the benefits arising out of the utilization of genetic resources. The KMGBF will be implemented by the more detailed instructions described in the monitoring framework (COP CBD 2022c). Countries will report on their progress on the indicators in the Monitoring Framework, which requires concrete data to be delivered that address the KMGBF's goals and targets. National reports on the Monitoring Framework will be due in 2026 and 2029 (COP CBD 2022b). At the CBD conference in 2030, the parties will evaluate whether this 3-year reporting period should be maintained or adjusted.

To fulfill the third objective of the CBD, goal C and target 13 of the KMGBF (table 1) require the parties to measure, for the first time, the benefits received from the use of genetic resources, associated traditional knowledge, and digital sequence information, as well as to facilitate access and increase benefits by 2050.

**Table 1.** Global goal and target text from the Kunming–Montreal Global Biodiversity Framework related to access and benefit-sharing.

Goal or target	Text
Goal C	The monetary and non-monetary benefits from the use of genetic resources and digital sequence information on genetic resources, and of traditional knowledge associated with genetic resources, as applicable, are shared fairly and equitably, including, as appropriate with Indigenous peoples and local communities, and substantially increased by 2050, while ensuring traditional knowledge associated with genetic resources is appropriately protected, thereby contributing to the conservation and sustainable use of biodiversity, in accordance with internationally agreed access and benefit-sharing instruments.
Target 13	Take effective legal, policy, administrative and capacity-building measures at all levels, as appropriate, to ensure the fair and equitable sharing of benefits that arise from the use of genetic resources and from digital sequence information on genetic resources, as well as traditional knowledge associated with genetic resources, and facilitating appropriate access to genetic resources, and by 2030, facilitating a significant increase of the benefits shared, in accordance with applicable international access and benefit-sharing instruments.

Access and benefit-sharing (ABS) is a legal concept under the third objective of the CBD that requires users of biodiversity to first ask permission before conducting research or development and, if necessary, to enter into an ABS agreement with the provider of the genetic resources. There are 196 parties to the CBD and 142 parties to the Nagoya Protocol (https://absch.cbd.int). Under the CBD and its Nagoya Protocol, the international focus in the past decades has been on legal implementation of these frameworks—rather than outcomes—and most attention has gone to developing legislative, administrative, policy, and compliance measures. Now, the big challenge for the parties is to develop a global approach and an indicator system to quantify outcomes—namely, the benefits from ABS agreements. In this respect, methodologies to measure monetary and non-monetary benefits need to be developed in order to account for the particularities of both types.

Non-monetary benefits, such as scientific training or information for conservation policy choices, are as important as monetary benefits, although they can be less tangible and more difficult to measure. Non-monetary benefits can have significant social, economic, and ecological impacts that can be more sustainable than the monetary benefits generated under ABS agreements (Ebert et al. 2023). It is therefore critical that non-monetary benefits be quantified both to gain visibility and, ultimately, to make well informed policy decisions. Because of the difficulty of measuring benefit-sharing, the KMGBF monitoring framework had placeholder indicators that were further developed in the 2022–2024 intersessional period. In a recent study for the CBD Secretariat, Muñoz-García and colleagues (2024) proposed a new headline indicator for non-monetary benefits within the monitoring framework: Goal C.2 is “Non-monetary benefits arising from applicable internationally agreed ABS instruments.”

Headline indicators are the high-level indicators for the KMGBF monitoring framework and reflect the CBD parties’ ambition of full implementation of the KMGBF by 2050. The study authors proposed six types of non-monetary benefits that will collectively enable the headline indicator (box 1), three of which can be collected at the global level.

Box 1. **Types of non-monetary benefits proposed to deliver on goal C.2.**

Indicators for three types of non-monetary benefits can be collected at the global level (C.2.1-3) and would be collected by the methodology presented here. The remaining three types would either be collected at the national level or could be collected in a new global platform (C.2.4-6). Modified from Muñoz-García and colleagues (2024).

**Global level**

**Indicator C.2.1** The number of research and development results arising from ABS instruments.

**Indicator C.2.2** The number of scientific publications relevant to conservation, sustainable use, food security, and public health arising from ABS instruments.

**Indicator C.2.3** The number of joint scientific publications arising from ABS instruments with authors from the provider country, where appropriate.

Note: *where appropriate* means that other ABS instruments, such as the High Seas Treaty, could decide not to use this indicator.

**National level**

**Goals C.2.4-6** The indicator is related to capacity building and development arising from ABS instruments (see KMGBF target 20).

The number of technology transfer events arising from ABS instruments.

The number of projects contributing to sustainable development arising from ABS instruments.

These indicators were based on non-monetary benefits described in the annex of the Nagoya Protocol that were amenable to global analysis:

- sharing of research and development results collaboration;
- cooperation and contribution in scientific research and development programs, particularly biotechnological research activities, where possible in the party providing genetic resources;
- access to scientific information relevant to conservation and sustainable use of biological diversity, including biological inventories and taxonomic studies; and
- research directed toward priority needs, such as health and food security, taking into account domestic uses of genetic resources in the party providing genetic resources.

These non-monetary benefit indicators enable a new connection between ABS and scientific research. They harness the robustness of the scientific publication ecosystem, accessible in public databases, where the data is curated and stored in standardized vocabularies and machine-readable fields, and exploit it for demonstrating the real-world outcomes and new knowledge generated by international scientific collaboration that results in non-monetary benefit-sharing (NMBS). In the present article, we propose a methodology to deliver global data on these three proposed indicators and quantify NMBS. The non-monetary benefits described in the present article are direct research outcomes from ABS agreements under the CBD or the Nagoya Protocol. Our findings will contribute to increased understanding, visibility, and quantification of non-monetary benefits, better understanding of the impact of international scientific collaboration, and support for parties in implementing one of the key headline indicators of the KMGBF. Furthermore, these approaches could be expanded and used by other international ABS instruments as foreseen by goal C and target 13 (table 1).

## Developing a database to support measuring non-monetary benefits at the global level

In order to assess the three globally-collected types of non-monetary benefits, we established a database of scientific publications in which an ABS permit (aka ABS agreement or mutually agreed terms; for the purpose of consistency, we will use the term *permit* throughout) is cited in the text of the publication (supplemental table S1). Because ABS permits were identified in the publication, the link to scientific research results and international collaborations—and, therefore a potential proxy for NMBS—is inherent. The database can provide quantification of the number of research results conducted under an ABS permit that were shared.

Our method requires access to full-text articles in order to search for ABS permit codes anywhere within the article. Below, we describe the steps employed to build the ABS permit publication database prototype.

### Step 1: List of IRCCs in the ABS Clearing House

**Figure 1.**
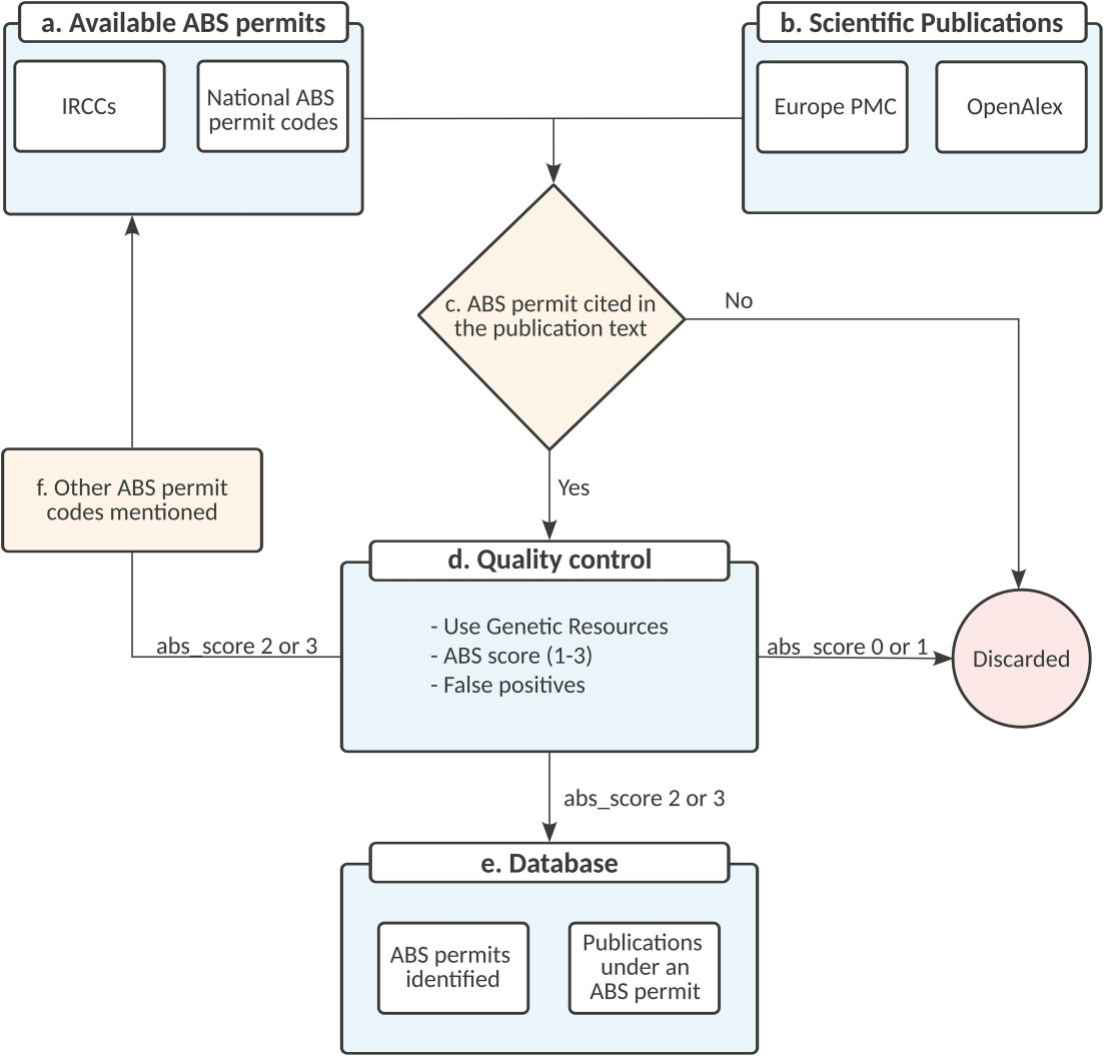
Flowchart describing the methodology and logic used to develop the database on non-monetary benefit-sharing from the use of genetic resources.

The search for ABS permits began with an assessment of internationally recognized certificates of compliance (IRCC) available in the ABS Clearing House. Countries are required under article 17.2 of the Nagoya Protocol (SCBD 2011) to upload their ABS permits or equivalents to the ABS Clearing House, which will subsequently automatically generate an IRCC. All IRCCs have unique and persistent identifiers that support the monitoring of use of genetic resources (SCBD 2011), and these identifiers can be downloaded in bulk from the ABS Clearing House (https://absch.cbd.int). We assumed that this established identifier would be readily cited by scientists together with their research outcomes (figure 1a). By the end of 2023, there were 5042 IRCC documents available, issued by 27 countries, although 5 countries represented around 90% of all IRCCs created in the ABS Clearing House: India (3496), France (750), Spain (190), Argentina (100), and Kenya (93).

All IRCC identifiers were searched for within scientific articles in the Europe PubMed Central database (figure 1b and 1c), a life science literature database with over 42 million abstracts and more than 9 million full text articles. The RESTful (for *representational state transfer*) APIs (application programming interface; https://europepmc.org/RestfulWebService) were employed and text-mining techniques used (Rosonovski et al. 2024). Out of 5042 IRCC in the ABS Clearing House, only 28 (0.6%) were cited in 39 research articles, which were added to the NMBS database. For example, although India has 3496 IRCCs, we detected eight publications only with Indian national ABS permit code. This is a vanishingly small number considering the total number of IRCCs and research articles available. On closer examination of the full-text articles, we noticed that researchers often cite national ABS permit codes (which are not standardized) rather than the IRCC codes.

### Step 2: List of national ABS permit codes in the ABS Clearing House

When the parties to the Nagoya Protocol submit their ABS permits to the ABS Clearing House to produce an IRCC, they should also include the reference number of the permit or its equivalent, to refer to any national ABS permit code (SCBD 2011). On the basis of the national ABS permits codes listed in the IRCCs, a supplemental list of national ABS permits codes from different countries was created to search for additional ABS permits mentioned in the Europe PubMed Central database (figure 1a). When ABS national permit codes were used for searching, the number of records increased by an order of magnitude to 560 (as of August 2024).

### Supplemental step: Direct requests of additional national ABS permit codes

Fifty four countries are party to the CBD but not the Nagoya Protocol and could have national permits under the CBD. In addition, not all parties comply with the IRCC mandate and could have national codes that were not identified. Therefore, to supplement the list of IRCCs and national ABS permit codes, the authors contacted all ABS national focal points by email requesting a list of their ABS permit codes (figure 1a). We also offered, in return, to provide any information discovered from the use of national ABS permit codes back to the country.

From a contact list of 178 national focal points obtained from the ABS Clearing House, only 10 parties replied. Australia and Costa Rica subsequently sent a list of all national ABS permit codes, which further increased the number of ABS permit codes in the database (13 and 102, respectively) and related publications (10 and 95, respectively). Additional lists of national ABS permit codes from the parties would likely improve the methodology and increase data on non-monetary benefit-sharing. If the method proposed in the present article becomes the official methodology for the KMGBF C.2 (NMBS) indicator, it is likely that more parties will become aware of the value of sharing ABS permit codes and the resulting data that can be made available to them as a result.

### Step 3: Quality control of the ABS permit publication database

When conducting *in situ* field research, many different kinds of permits can be required at the national level and be cited in the publication, some of which may not be ABS relevant. To ensure a high-quality data set and a maximum level of certainty that the database contains publications that cite ABS permits, every result from the Europe PubMed Central was checked manually to avoid false positives and conduct quality checks (figure 1d).

The database contains two main tables. The “abs_permits” table includes fields about ABS permit codes cited in the article, the country issuing the ABS permit, and any ABS national authority mentioned in the publication. The “research_article” table included fields such as unique article ids (PMID, PMCID, doi) and features including the title, the OpenAlex Topic ID, the author’s full name, institutional affiliations, the publication year, the location of where the ABS permit is mentioned, and the abstract. During the manual curation process, two flags were added: “has_gr,” which indicates the article clearly describes accessing genetic resources and the provider country is recorded, and “is_false_positive,” which indicates the article text has a string similar to a putative ABS permit code, but, on manual evaluation, actually refers to something else (not directly related to ABS).

During the manual evaluation of the scientific articles, some permit codes not present in the initial list of permits were additionally identified as possible ABS permits. These codes and their issuing countries were subsequently annotated in the database, and an additional evaluation was conducted to assess whether those new codes represented actual ABS permits (figure 1f). The evaluation involves verifying any institutions mentioned in the publication against the national ABS authority of the issuing country, as per the ABS Clearing House or the national website.

Each ABS permit code was assigned an “abs_score,” a whole number ranging from 0 to 3. If a presumptive ABS code did not represent an ABS permit during the quality control analysis, the reviewer (GNV or a student assistant) assigned an ABS score of 0, and the article was discarded from further analysis. If a code could be verified as a true ABS permit, the reviewer assigned a score of 3. ABS scores of 1 were assigned to codes in publications that use genetic resources, but they lack sufficient information about the issuing country to either confirm or reject as an ABS permit code (low quality). Scores of 2 were given to codes where confirmation was not possible (i.e., because a list of permit codes was not available from the national authority), although the publication text indicates it is an ABS permit code (medium quality).

The data set presented in the present article contains all records where the ABS permit code has a likely or verifiable ABS agreement (“abs_score = 2 or 3”) and the article that cites the permit actually accessed genetic resources (“has_gr = 1”). Supplemental table S2 shows the number of records that correspond to every abs_score. All false positives were excluded.

### Step 4: Author affiliations

Using the author affiliation information (the location of the authors via their institutional affiliations) listed in the publication, we determined whether scientific collaboration took place and whether the coauthors were in the country that provided the genetic resource using the methodology described in (Lange et al. 2021). To improve accuracy, the author tables were reviewed manually to reduce errors (Sebo et al. 2021).

### Step 5: Assignment of publications to a specific topic

The subject matter topic for each publication in our database was obtained from the OpenAlex system using its API (https://docs.openalex.org/api-entities/topics). This is an automated system that uses fine-tuned large language models to classify publications, considering available information such as title, abstract, journal name, and citations, and it was built on top of the methodology made by the Centre for Science and Technology Studies at Leiden University (Waltman and Van Eck 2012, Priem et al. 2022, OpenAlex 2024), in Leiden, in the Netherlands. Each publication in the OpenAlex database includes a main topic structured in higher categories such as subfield, field, and domain. To determine the publications relevant to the proposed priority research areas, two reviewers (GNV and Davide Faggionato; see the acknowledgments) independently mapped each level of the subfield category into the four categories deemed priority areas by the CBD: conservation, sustainable use, public health, or food security, as well as to other for all other fields (supplemental file S1, supplemental table S4). A third colleague reviewed cases in which two different labels where assigned (Aylin Haas; see the acknowledgments section).

## Non-monetary benefits assessed at the global level

Three types of non-monetary benefits can be quantified with the proof-of-principle data set established in this work and made publicly available (see the acknowledgements below). NMBS data can be collected globally, disaggregated nationally, and made available to countries for use in their national reports.

**Figure 2.**
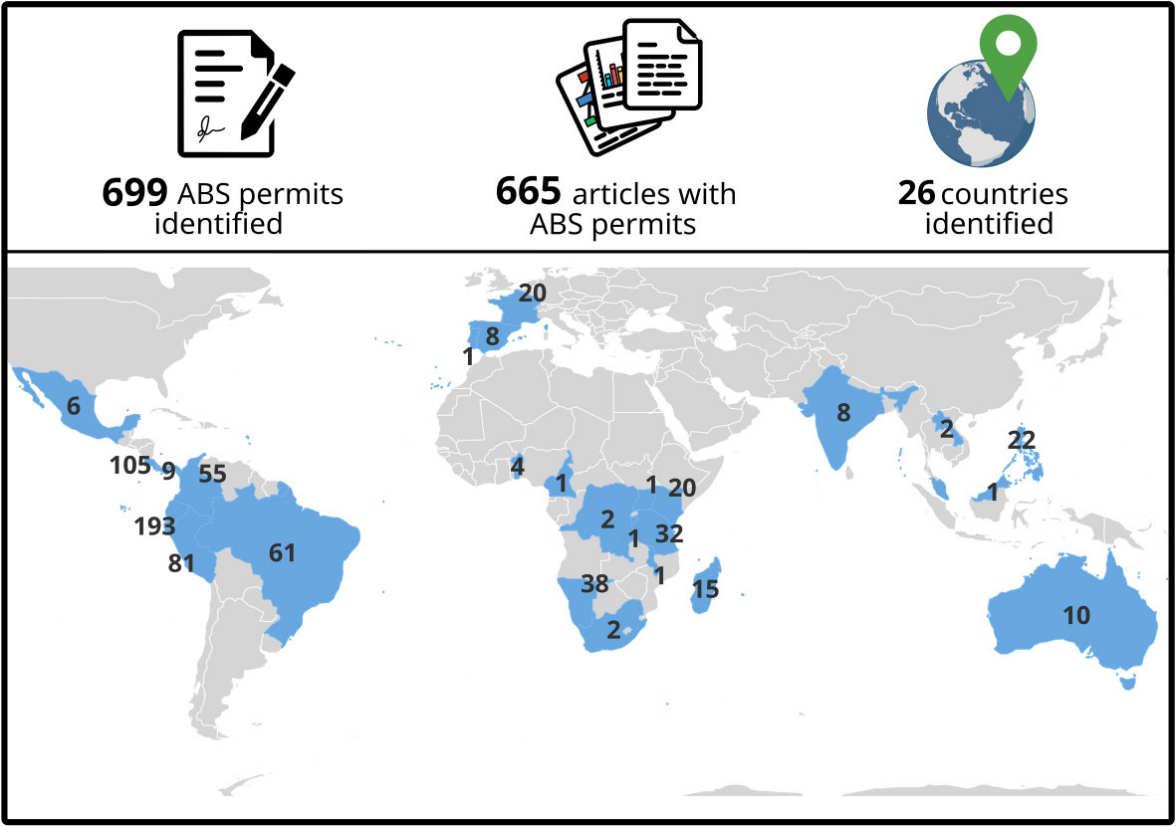
Composition and geographical distribution of the ABS permit database created as part of the ET-NMBS project. These represent the sharing of 665 research and development results (indicator C.2.1). Polygons with numbers represents countries where an ABS permit was mentioned in the scientific articles and the value inside polygons indicates the number of articles per country. Empty polygons represents countries with no information in our database. 13 articles identified both IRCC and national ABS permit codes. Only ABS permit codes with abs_score 3 and 2 were used for this figure. See supplemental tables S2 and S3 for additional details. Icons created with BioRender.com.

**Figure 3.**
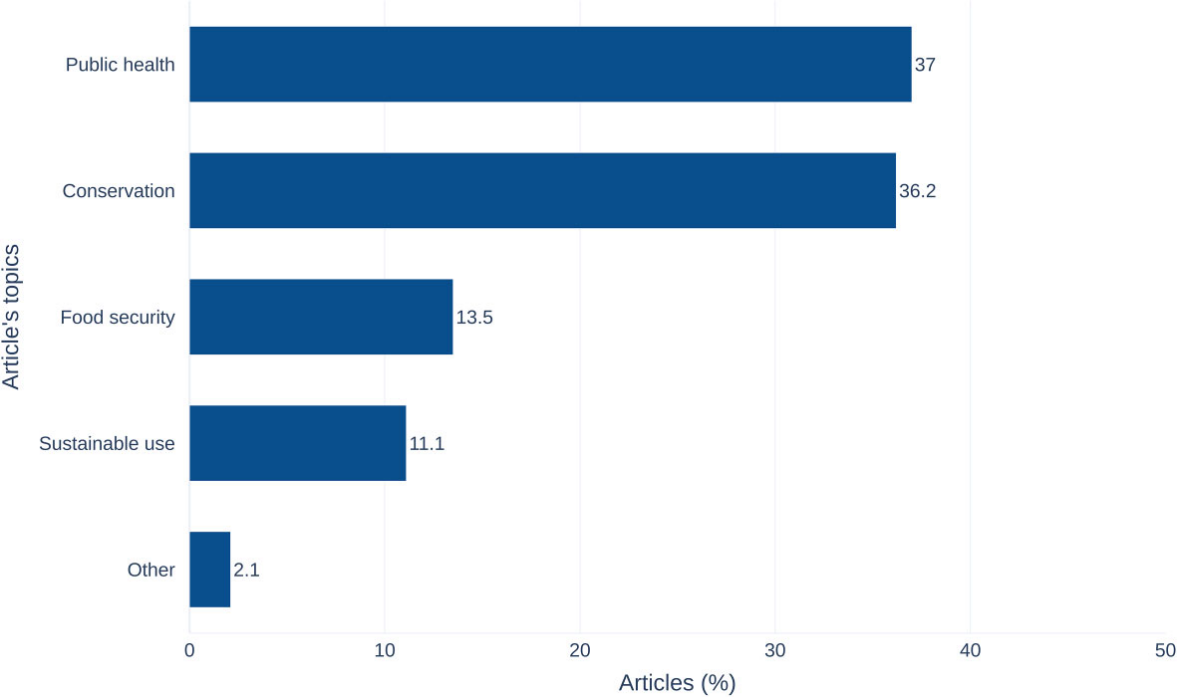
The percentage of articles per priority research area (type of non-monetary indicator C.2.2). 77 subfields from OpenAlex identified in 665 publications in the database were mapped into five categories: conservation, public health, food security, sustainable use, and other. The mapping of subfields is available in supplemental table S3.

We successfully created a curated database connecting 699 ABS permits from 26 countries cited in 665 research articles (figure 2, table S4). According to our analysis, the number of publications that cite an ABS permit has increased over time, with the oldest publication recorded in our database published in 2010 (figure 3; note: the CBD came into force in 1992 and the Nagoya Protocol in 2014). The publicly available database is still growing as new ABS permit code patterns and new examples from countries arrive, and a new website to host these data is under development. But we can already show some trends in some types of non-monetary benefit-sharing via scientific publications.

**Figure 4.**
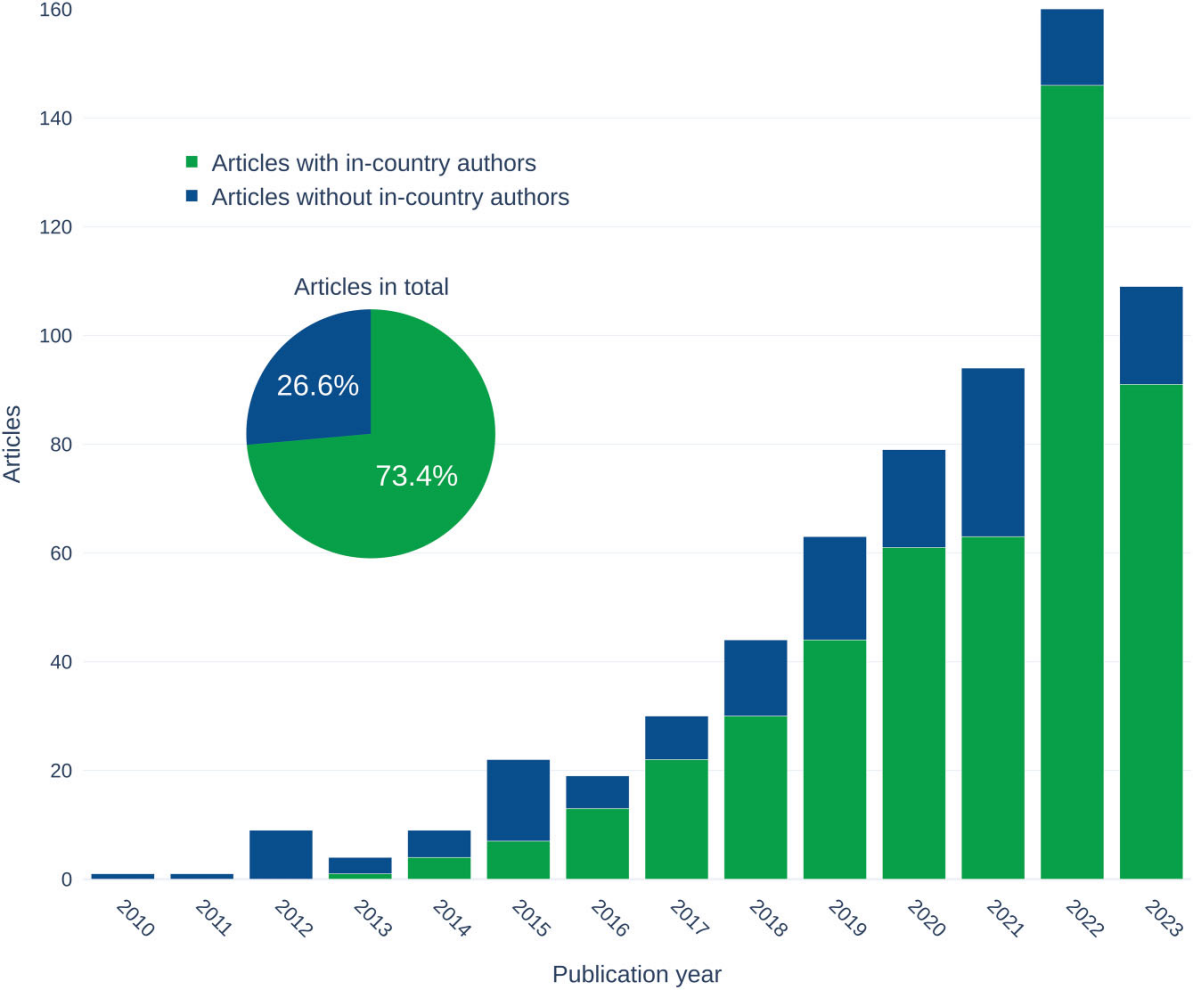
The number of publications (total bar height) over time in the NMBS database with (bottom bar) or without (top bar) in-country authors (type of non-monetary indicator C.2.3). An in-country author is a coauthor whose geographical affiliation is located in the country that provided access to the genetic resource used in the research article. In total, 73.4% of the 665 articles in the database have in-country authors (pie chart inset).

### Indicator C.2.1: The number of research and development results arising from ABS instruments

In this approach, the 665 scientific publications are used as a proxy for research and development results. Ecuador has the highest number of ABS-permit-citing publications (supplemental figure S1). Ecuador facilitates access to genetic resources when they are used exclusively for noncommercial research purposes through easy-to-execute framework contracts (Sirakaya 2019), which could explain why the highest number of publications in the database come from this country (i.e., because access is facilitated). The majority of IRCCs in the database are from France, which is the country with the second highest amount of IRCCs available in the ABS Clearing House. Although India has the most IRCCs available, only eight Indian national permits codes, rather than IRCCs, could be found. More research and discussion with the Indian competent national authority is needed to understand this discrepancy. Costa Rica is the country with the highest number of unique ABS permits which could be because the authorities shared a full list of all national ABS permits leading to a complete dataset to search for.

### Indicator C.2.2: The number of scientific publications arising from ABS instruments relevant to conservation, sustainable use, food security, and public health

Publications are more than just research results. They also contribute to significant societal needs and can show where and how ABS has had an impact. The majority of publications on the use of genetic resources are relevant to public health (37%), followed by conservation (36.2%), food security (13.5%), and sustainable use (11.1%; figure 3). These results show how the database can contribute to quantifying non-monetary benefits such as “research directed toward priority needs, such as health and food security, taking into account domestic uses of genetic resources in the party providing genetic resources,” per the annex to the Nagoya Protocol. For example, Ecuador, the country with the highest number of ABS-permit-citing publications (193), shows a similar trend, with 31.6% relevant to public health, 29% relevant to conservation, followed by 21.8% of publications relevant to sustainable use, and 29% of publications relevant to food security (supplemental figure S1). Although Ecuador is a biodiversity hotspot, there is still significant research on public health needs. Biodiversity-related research, such as new species descriptions does not overly dominate the research conducted under ABS permits. This might suggest that the scientific research in Ecuador is reflective of overall scientific research trends or priority setting at the national level.

### Indicator C.2.3: The number of joint scientific publications arising from ABS instruments with authors from the provider country, where appropriate

By analyzing the authorship in the database as a proxy for international collaboration in publications involving ABS obligations, we were able to assess whether researchers in the providing country (the source of the genetic resource) were involved in the research results published. A total of 73.4% of the articles in the database have at least one in-country author, which means that, in most ABS cases, a researcher in the country that provided the genetic resource was substantially involved in the research. An in-country research partner is often a requirement of ABS national legislation (e.g., in Brazil), but not all ABS-regulating countries require the participation of local partners in ABS permits (Sirakaya 2019, Heinrich et al. 2020). This depends on national legislation, so publications without in-country authors do not necessarily indicate that ABS responsibilities were not fulfilled. Indeed, good scientific practice requires that only authors who made a significant research contribution should be listed. Figure 4 shows changes over time, with more publications with in-country authors in recent years. This could suggest that ABS frameworks are having a positive impact on coauthorships and international collaboration, although causality cannot be determined for this change.

## Policy implications: Recommendations for more effective NMBS information collection at the global level

The prototype and methodology for assessing these non-monetary benefit types at the global level could be further improved by complementary efforts by ABS competent national authorities, the scientific community, journal editors, and the ABS Clearing House.

### Recommendation 1: Require users to cite IRCCs or ABS permits in scientific publications

In the non-monetary benefit database, just 39 research articles cited IRCCs, which is a small number considering the requirements to produce an IRCC under the Nagoya Protocol. Researchers most often cited national ABS permit codes that are not standardized across countries rather than IRCC codes. This makes it more challenging to link research and development results via research articles to the ABS agreements at the global level and impedes future reporting of the proposed non-monetary benefit indicators (box 1). To improve the citation of IRCCs in research articles, three improvements are necessary:

1. Countries must publish their ABS permits in the ABS Clearing House, which generates IRCCs codes that should be made available to researchers to be cited, in order to robustly link research results with IRCCs codes, which is an obligation under the Nagoya Protocol.
2. In the interim, countries should provide their national ABS permit codes to the ABS Clearing House or, if easier, to the authors, to further optimize the methodology described in the present article.
3. Countries could require in the ABS permit an obligation to cite the IRCCs (or ABS permits codes) in all publications related to the authorized use of genetic resources or associated traditional knowledge.

### Recommendation 2: Standardize ABS permit citation practices

Our dataset shows that researchers cite their ABS agreements in different locations throughout their publication (supplemental table S5). 47.7% of the articles cited the ABS permit in their “Material and Methods” section, whereas 29.1% cited their ABS permit in their “Acknowledgments” section; the rest used a variety of sections. These varied locations make the automated detection and prediction of what is an ABS permit more difficult. ABS citation practices need to become more standardized, and leadership from scientific journals is needed. Improvement in ABS permit citation will make it easier to quantify non-monetary benefits from the use of genetic resources and associated traditional knowledge.

### Recommendation 3: Journals should consider developing benefit-sharing policies

Not all scientists that have obtained an ABS permit routinely cite it in their research results. And, in some cases, scientists might not have followed ABS procedures and national legislation. To this end, some journals have put in place benefit-sharing-related policies such as the journals *Plants* (MDPI 2024), and *Molecular Ecology and Molecular Ecology Resources* and the publisher PLOS (2024), which require authors to certify that they have followed ABS laws per the CBD and the Nagoya Protocol as a condition for publication (Marden et al. 2021). Some journals also encourage authors to include a new section in their article called “Data Accessibility and Benefit-Sharing” to disclose the benefits shared. These initiatives contribute to solving the problem of invisibility of non-monetary benefit-sharing, but there is still work to do toward the standardization of best practices for ABS in scientific publications and therefore a current limitation to this global approach.

### Recommendation 4: Develop a new standardized global reporting system for users to self-report benefits shared

Another complementary approach for benefit-sharing reporting would be to develop a global reporting system for non-monetary benefits especially for those benefits not currently captured globally or for closed-access publications (see box 1, national level). This would be a new system that would gather information in a centralized manner that can subsequently be disaggregated by country. This reporting tool would enable users of genetic resources and their associated traditional knowledge to report on the benefits shared in their work under an ABS agreement. A global standardized repository for non-monetary benefit-sharing reports would streamline the process of gathering data on non-monetary benefits and would enable a link to an IRCC, a benefit-sharing report and a publication, further strengthening the interconnectivity of the ABS ecosystem. Such a system would decrease the national burden on ABS reporting while still providing for disaggregation by country and automated notifications of new non-monetary benefits back to provider countries. It would also be an efficient use of financial resources because it involves the development of a single database instead of one per country. Nevertheless, such an approach would require the development of a common framework and methodology on how to report on non-monetary benefits. Importantly, the final determination of whether the benefit-sharing was sufficient and fulfills the agreed conditions under the mutually agreed terms would still remain with the providing party. User reporting of benefits shared can also be seen as a chance to create data on benefit-sharing and increase the responsibility for users to provide benefits that align with the overarching CBD principle that ABS should contribute to conservation and sustainable use of biodiversity.

Global reporting by users could also be linked with the global reporting standard from the Global Reporting Initiative (GRI; GRI 2024). The GRI Reporting Standard 101 on Biodiversity is a framework that guides organizations in reporting their impacts on biodiversity. The standard offers guidance on disclosing biodiversity-related information in sustainability reports. GRI reporting requires disclosure of information on the organization's principles and activities. Voluntary user reporting as described under the GRI standards for biodiversity could be used as a tool to compile benefits shared and develop a centralized platform.

## Conclusions

We propose in the present article a new global methodology to quantify non-monetary benefit-sharing from scientific publications that cite ABS permits. The database prototype can quantify three types of non-monetary benefits and will continue to be developed until 2025 through project funding. This methodology will be employed to provide initial data on NMBS to countries and can be expanded to include other ABS instruments if the parties of those instruments choose to. Global approaches free up capacities for authorities to focus on national reports and monetary benefits, as well as other types of non-monetary benefits not captured globally. A new centralized reporting database for users to report on benefits shared would provide a complementary global approach to capturing non-monetary benefits. All data collected at the global level will subsequently be disaggregated and made available to countries for their use in their national report. These approaches and the prototype can be further expanded into other kinds of non-monetary benefit-sharing such as joint intellectual property rights using other data types and databases, as well as being potentially useful for digital sequence information (Scholz et al. 2021).

Efforts from both providers and users will improve the ability to assess non-monetary benefits from the use of genetic resources and their associated traditional knowledge. The parties to the Nagoya Protocol and the CBD have to take measures to make information about ABS agreements more accessible to the research community and scientific practices for citing IRCCs or ABS permits codes in scientific publications need to be better standardized. Scientific journals and the research community should play a leadership role in this issue.

## Supplementary Material

biae132_Supplemental_Files

## Author Biography

Genuar Nunez-Vega is a research associate in the Science Policy and Internationalization Department at the Leibniz Institute DSMZ and a PhD student at the Technical University of Braunschweig, in Braunschweig, Germany. Lorenz Christian Reimer is one of the joint heads of the Department of Bioinformatics at the Leibniz Institute DSMZ, in Braunschweig, Germany. Jörg Overmann is the scientific director of the Leibniz Institute DSMZ and a professor at the Technical University of Braunschweig, in Braunschweig, Germany. Amber Hartman Scholz (amber.h.scholz@dsmz.de) is the head of the Department of Science Policy and Internationalisation at the Leibniz Institute DSMZ, in Braunschweig, Germany.

## References

[bib1] [COP CBD] Conference of the Parties to the Convention on Biological Diversity . 2022a. Kunming–Montreal Global Biodiversity Framework. United Nations Environment Programme. Decision no. CBD/COP/DEC/15/4.. www.cbd.int/doc/decisions/cop-15/cop-15-dec-04-en.pdf

[bib2] [COP CBD] Conference of the Parties to the Convention on Biological Diversity . 2022b. Mechanisms for Planning, Monitoring, Reporting, and Review. United Nations Environment Programme. Decision no. CBD/COP/DEC/15/6. www.cbd.int/doc/decisions/cop-15/cop-15-dec-06-en.pdf

[bib3] [COP CBD] Conference of the Parties to the Convention on Biological Diversity . 2022c. Monitoring Framework for the Kunming–Montreal Global Biodiversity Framework. United Nations Environment Programme. Decision no. CBD/COP/DEC/15/5. www.cbd.int/doc/decisions/cop-15/cop-15-dec-05-en.pdf

[bib6] Ebert AW, Engels JMM, Schafleitner R, Hintum TV, Mwila G. 2023. Critical review of the increasing complexity of access and benefit-sharing policies of genetic resources for genebank curators and plant breeders: A public and private sector perspective. Plants 12: 2992. 10.3390/plants12162992 37631201 PMC10459714

[bib4] [GRI] Global Reporting Initiative . 2024. Transparency standard to inform global response to biodiversity crisis. Global Reporting Initiative (25 January 2024). www.globalreporting.org/news/news-center/transparency-standard-to-inform-global-response-to-biodiversity-crisis

[bib7] Heinrich M et al. 2020. Access and benefit sharing under the Nagoya Protocol: *Quo Vadis*? Six Latin American case studies assessing opportunities and risk. Frontiers in Pharmacology 11: 765. 10.3389/fphar.2020.00765 32581783 PMC7294742

[bib8] Marden E et al. 2021. Sharing and reporting benefits from biodiversity research. Molecular Ecology 30: 1103–1107. 10.1111/mec.15702 33159357

[bib9] [MDPI] Multidisciplinary Digital Publishing Institute . 2024. Instructions for authors: Research involving plants. MDPI. www.mdpi.com/journal/plants/instructions

[bib10] Muñoz-García M, Lago A, Scholz, eds AH. 2024. Access and Benefit-Sharing Indicators for the Kunming–Montreal Global Biodiversity Framework. Convention on Biological Diversity. Report no. CBD/SBSTTA/26/INF/12. www.cbd.int/doc/c/6920/4e1e/8a6ba925279ea19033eb8ed2/sbstta-26-inf-12-en.pdf

[bib10a] Lange M, Alako BTF, Cochrane G et al. 2021. Quantitative monitoring of nucleotide sequence data from genetic resources in context of their citation in the scientific literature. GigaScience 10. 10.1093/gigascience/giab084 PMC8716361 34966925

[bib11] OpenAlex . 2024. OpenAlex: End-to-end process for topic classification. Google Docs. https://docs.google.com/document/d/1bDopkhuGieQ4F8gGNj7sEc8WSE8mvLZS/edit

[bib12] [PLOS] Public Library of Science . 2024. Best practices in research reporting. PLOS. https://journals.plos.org/plosntds/s/best-practices-in-research-reporting

[bib13] Priem J, Piwowar H, Orr R. 2022. OpenAlex: A fully open index of scholarly works, authors, venues, institutions, and concepts. arXiv 2205.01833. 10.48550/ARXIV.2205.01833

[bib14] Rosonovski S et al. 2024. Europe PMC in 2023. Nucleic Acids Research 52: D1668–D1676. 10.1093/nar/gkad1085 37994696 PMC10767826

[bib5] [SCBD] Secretariat of the Convention on Biological Diversity . 2011. Nagoya Protocol on Access to Genetic Resources and the Fair and Equitable Sharing of Benefits Arising from Their Utilization to the Convention on Biological Diversity: Text and Annex. SCBD.

[bib5a] Scholz AH, Matthias L, Pia H, Paul O, Ibon C, Guy C, Jens F. 2021. Myth-busting the provider-user relationship for digital sequence information. GigaScience 10. 10.1093/gigascience/giab085 PMC8716360 34966927

[bib5b] Sebo P, Sylvain DL, Nathalie V. 2021. Accuracy of pubmed-based author lists of publications and use of author identifiers to address author name ambiguity: A cross-sectional study. Scientometrics 126: 4121–4135. 10.1007/s11192-020-03845-3

[bib15] Sirakaya A . 2019. Balanced options for access and benefit-sharing: Stakeholder insights on provider country legislation. Frontiers in Plant Science 10: 1175. 10.3389/fpls.2019.01175 31632420 PMC6781883

[bib16] Waltman L, Van Eck NJ. 2012. A new methodology for constructing a publication-level classification system of science. Journal of the American Society for Information Science and Technology 63: 2378–2392. 10.1002/asi.22748

