# Supplementary material for: A new indicator for the Kunming–Montreal Global Biodiversity Framework: Capturing non-monetary benefit data from access and benefit-sharing agreements
Source: Bioscience. 2025 Feb 19;75(4):298–306. doi: 10.1093/biosci/biae132 (PMC12016788; doi:10.1093/biosci/biae132)
Supplement: biae132_Supplemental_Files [file biae132_supplemental_files.zip › supplementary_file_1.docx]

**Supplementary File 1**

**Re-mapping the OpenAlex subfields into four priority areas of research**

One of the indicators of non-monetary benefits proposed for the KMGBF monitoring framework is “Number of scientific publications relevant to conservation, sustainable use, food security and public health arising from ABS instruments”. The classification of publications can be built off of the OpenAlex database. OpenAlex created Topics, an automated system that uses fine-tuned Large Language Models (LLM) to classify publications, and it is described in the document “[OpenAlex: End-to-End Process for Topic Classification](https://docs.google.com/spreadsheets/d/1v-MAq64x4YjhO7RWcB-yrKV5D_2vOOsxl4u6GBKEXY8/edit?gid=983250122#gid=983250122)”. Topics are structured in a hierarchy of 4 domain, 26 fields, 252 subfields, and 4616 topics names.

The OpenAlex Topics were categorized into conservation, sustainable use, food security and public health using the expertise of the reviewers. A definition of each of these priority research areas is at the end of the document.

**Instructions**

- Reclassify each label in the subfield category (252 labels in total) into conservation, sustainable use, food security, public health or other (5 new labels).
- Assign the most likely new label to subfields according to your expertise but only one (single label classification).
- In case of ambiguity, the category topics names may be helpful to describe in more detail what the publication is about and guide the classification. Fields and domain can also be used, but the focus is on subfields.
- Use the file “OpenAlex_topic_mapping_table.xlsx” for this task, the tab “final_topic_field_subfield_tabl” has all the OpenAlex Topics, and the tab “subfields” has the reviewer mappings.

**Definitions**

**Conservation**

Refers to the conservation of the biological diversity. According to the text of the CBD “Biological diversity” *means the variability among living organisms from all sources including, inter alia, terrestrial, marine and other aquatic ecosystems and the ecological complexes of which they are part; this includes diversity within species, between species and of ecosystems* (UN 1992).

**Sustainable use**

Refers to the sustainable use of biodiversity. According to the text of the CBD “Sustainable use” *means the use of components of biological diversity in a way and at a rate that does not lead to the long-term decline of biological diversity, thereby maintaining its potential to meet the needs and aspirations of present and future generations* (UN 1992).

**Public health**

According to the text of the Constitution of the World Health Organization (https://apps.who.int/gb/bd/PDF/bd47/EN/constitution-en.pdf?ua=1), *Health is a state of complete physical, mental and social well-being and not merely the absence of disease or infirmity*. In the WHO world report of 1998 public health is defined as “*The art of applying science in the context of politics so as to reduce inequalities in health while ensuring the best health for the greatest number*” (WHO 1998).

**Food security**

According to the Rome Declaration on World Food Security “Food security exists when all people, at all times, have physical, [social] and economic access to sufficient, safe and nutritious food which meets their dietary needs and food preferences for an active and healthy life” (FAO 1996).

**References**

Azari R, Borisch B. What is public health? a scoping review. Arch Public Health. 2023 May 10;81(1):86. doi: 10.1186/s13690-023-01091-6. PMID: 37165370; PMCID: PMC10170773.

FAO. 1996. Rome Declaration on World Food Security and World Food Summit Plan of Action : World Food Summit, 13-17 November 1996, Rome, Italy. https://www.fao.org/4/w3613e/w3613e00.htm

United Nations. 1992. Convention on Biological Diversity United Nations.

World Health Organization. The World health report: 1998: life in the 21st century: a vision for all: report of the Director-General. Geneva: World Health Organization; 1998.


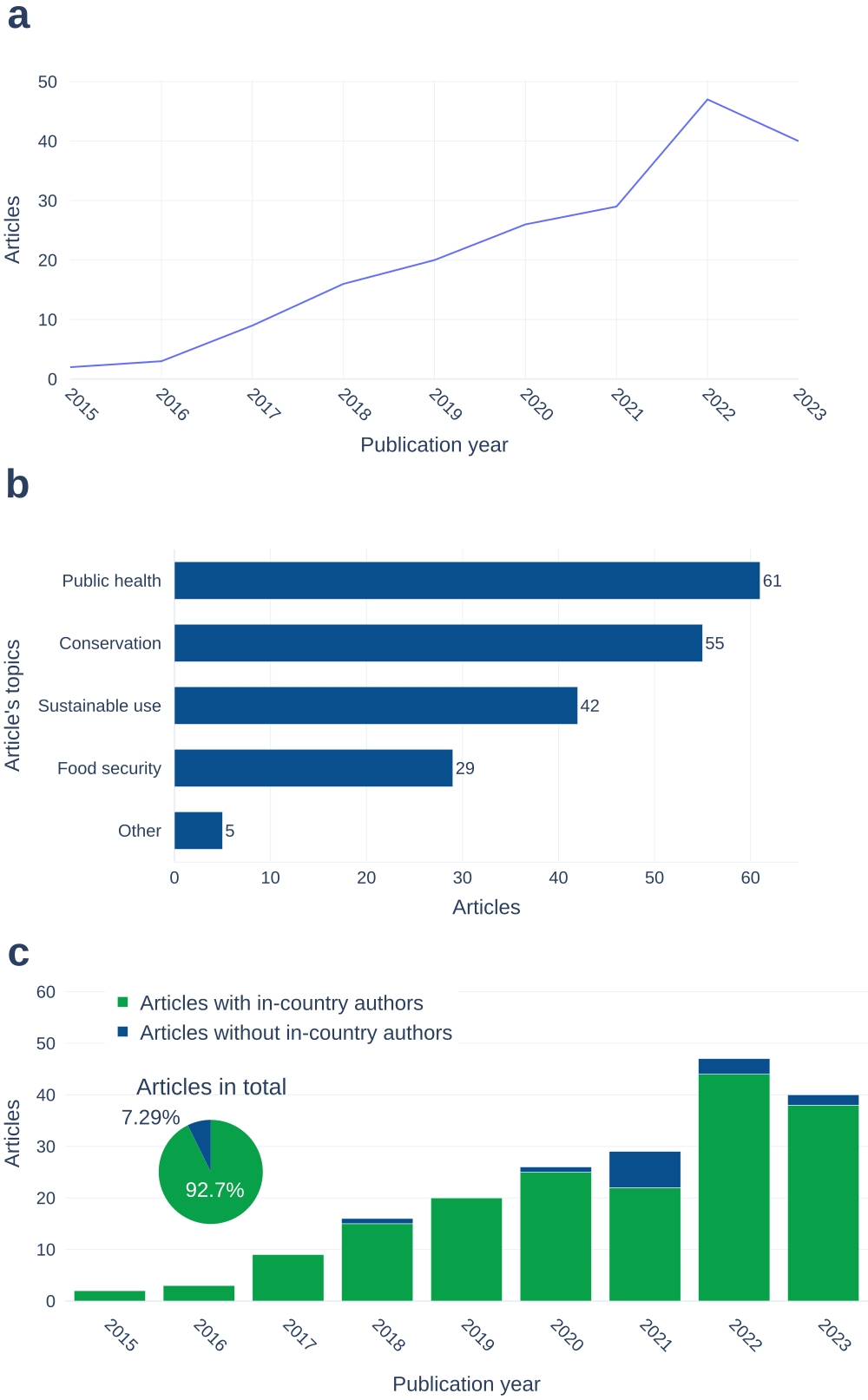


Supplementary Figure 1. Database information on non-monetary benefit-sharing indicators for Ecuador. (a) Number of research and developments results in Ecuador over time. (b) Number of scientific publications relevant to priority areas. (c) Number of joint scientific publications with authors from the provider country over time and in total (pie chart inserted).

Supplementary table 2: Database composition based on ABS quality score of the ABS permit codes. The rows show the number of ABS permits, number of publications, and number of countries for high, medium, and low-quality scores.

|  | **High quality score** | **High+Medium quality score** | **High+Medium+Low quality score** | **High+Medium+Low quality score+Discarded** |
| --- | --- | --- | --- | --- |
| **Number of ABS permits** | 598 | 669 | 937 | 1193 |
| **Number of research articles** | 631 | 665 | 701 | 740 |
| **Number of countries** | 20 | 26 | 37 | 50 |

*Supplementary table 4: Number of publications, Internationally Recognized Certificate of Compliance (IRCCs) and national ABS permit numbers by country in the database. The total number of Publications is higher than the number of total publications reported (665) since some publications use permits from more than one country.*

| **Country** | **Publications** | **IRCCs** | **National ABS permits** |
| --- | --- | --- | --- |
| Australia | 10 | 0 | 13 |
| Benin | 4 | 0 | 3 |
| Brazil | 61 | 0 | 73 |
| Burundi | 1 | 0 | 3 |
| Cameroon | 1 | 0 | 1 |
| Colombia | 55 | 0 | 73 |
| Costa Rica | 105 | 0 | 117 |
| Democratic Republic of the Congo | 2 | 0 | 2 |
| Ecuador | 193 | 0 | 89 |
| France | 20 | 19 | 5 |
| India | 8 | 0 | 8 |
| Kenya | 20 | 0 | 13 |
| Madagascar | 15 | 0 | 33 |
| Malawi | 1 | 0 | 1 |
| Malaysia | 1 | 0 | 2 |
| Mexico | 6 | 2 | 0 |
| Namibia | 38 | 0 | 47 |
| Panama | 9 | 1 | 17 |
| Peru | 81 | 1 | 71 |
| Portugal | 1 | 0 | 7 |
| South Africa | 2 | 1 | 6 |
| Spain | 8 | 7 | 5 |
| Tanzania | 32 | 0 | 52 |
| The Lao People's Democratic Republic | 2 | 1 | 1 |
| The Philippines | 22 | 0 | 25 |
| Uganda | 1 | 0 | 1 |
| **Total** | **699** | **32** | **668** |
